# Severe oral mucositis relating to pain and worse oral condition among patients with solid tumors undergoing treatment with FOLFIRI and 5-FU: a retrospective study

**DOI:** 10.1590/1516-3180.2021.0522.R1.22112021

**Published:** 2022-08-08

**Authors:** Laura Costa de Almeida, Bernardo da Fonseca Orcina, Aloizio Premoli Maciel, Dayanne dos Santos, Brena Rodrigues Manzano, Paulo Sérgio da Silva Santos

**Affiliations:** I DDS. Dental Surgeon, Private Practice, Bauru (SP), Brazil.; II DDS. Master’s Student, Department of Surgery, Stomatology, Pathology and Radiology, Faculdade de Odontologia de Bauru (FOB), Universidade de Sao Paulo (USP), Bauru (SP), Brazil.; III DDS, MSc. Doctoral Student, Department of Surgery, Stomatology, Pathology and Radiology, Faculdade de Odontologia de Bauru (FOB), Universidade de Sao Paulo (USP), Bauru (SP), Brazil.; IV DDS. Master’s Student, Department of Surgery, Stomatology, Pathology and Radiology, Faculdade de Odontologia de Bauru (FOB), Universidade de Sao Paulo (USP), Bauru (SP), Brazil.; V DDS, MSc. Doctoral Student, Department of Surgery, Stomatology, Pathology and Radiology, Faculdade de Odontologia de Bauru (FOB), Universidade de Sao Paulo (USP), Bauru (SP), Brazil.; VI DDS, PhD. Associate Professor, Department of Surgery, Stomatology, Pathology and Radiology, Faculdade de Odontologia de Bauru (FOB), Universidade de Sao Paulo (USP), Bauru (SP), Brazil.

**Keywords:** Stomatitis, Dental service, hospital, Drug therapy, Oral health, Oral medicine, Pathology, oral, Chemotherapy, Oral mucositis, Stomatology

## Abstract

**BACKGROUND:**

There is a need for studies that correlate the severity of oral mucositis (OM) with chemotherapy protocols, transient myelosuppression and oral health.

**OBJECTIVE:**

To analyze the severity of OM among individuals with solid tumors during hospitalization and its correlation with the type of chemotherapy, myelosuppression and oral health condition.

**DESIGN AND SETTING:**

Retrospective study at a public hospital in Bauru, state of São Paulo, Brazil, that is a regional referral center.

**METHODS:**

Individuals diagnosed with solid malignant tumors who received chemotherapy during hospitalization for completion of the antineoplastic treatment cycle or who presented complications resulting from this were assessed.

**RESULTS:**

Twenty-eight individuals (24.3%) manifested some degree of OM. The most prevalent degrees of OM according to the World Health Organization (WHO) and modified WHO classification were grades 2 (11.3%) and 5 (4.3%), respectively. It was observed that the higher the OM-WHO (P < 0.001; r = 0.306) and modified OM-WHO (P < 0.001; r = 0.295) classifications were, the greater the oral pain reported by the individuals was. Presence of mucositis in the upper lip and buccal mucosa contributed to increased severity of OM and worsening of swallowing during hospitalization. Thus, severe OM was associated with use of the FOLFIRI protocol (folinic acid, fluorouracil and irinotecan).

**CONCLUSION:**

Individuals with tumors who presented severe OM had greater severity of oral pain and worse oral health. Use of the FOLFIRI protocol was associated with higher prevalence of severe OM, while use of 5-fluorouracil (5-FU) was correlated with worse oral condition.

## INTRODUCTION

Oral mucositis (OM) is an inflammatory process caused by the cytotoxic effect of antineoplastic treatments (AnTs). This adverse effect has a large impact on individuals following certain chemotherapy protocols for treatment of solid tumors, such as cytarabine, 5-fluorouracil (5-FU) and alkylating and platinum derivatives.^1,2^ During these chemotherapy protocols, OM affects both the mouth and the entire gastrointestinal tract.^3^

OM occurs mainly in the mucosa and is not keratinized in the form of erythematous lesions and/or ulcers. It may or may not be associated with edema, burning and intense pain. These signs and symptoms significantly impair the quality of life of individuals under hospitalization and affect speech, swallowing and chewing. Therefore, worse nutritional status often indicates enteral or parenteral nutrition, use of systemic analgesics, increased inpatient hospital time and interruption of AnT.^1,4,5^

Certain forms of chemotherapy, besides causing OM, lead to transient myelosuppression and blood pancytopenia. Thus, aside from direct health-related problems, these cause decreased leukocyte counts and are associated with a high risk of opportunistic infections and exacerbation of infections.^6,7^

In the literature, few studies have correlated the severity of OM with the chemotherapy protocols used, its relationship with transient myelosuppression or the oral health of individuals under hospitalization.^1,7–9^ In addition, there is a lack of studies comparing the severity of OM with the use of oral health indicators and pain assessment by means of an OM-related visual analogue scale (VAS). Moreover, the relationship of these variables with transient myelosuppression has not been assessed.

## OBJECTIVE

The aim of this study was to assess the severity of OM in relation to the type of chemotherapy, oral health condition and myelosuppression among individuals with solid tumors who had been hospitalized for treatment.

## METHODS

### Characterization of the study and sample, and ethical matters

This retrospective cross-sectional study included individuals diagnosed with solid malignant tumors who were undergoing chemotherapy and who had been hospitalized for a single cycle or who had been hospitalized due to oral complications from the previous chemotherapy cycle utilized, at a public hospital between 2015 and 2017.

Patients for whom insufficient data were available in the electronic medical records or who were not receiving dental care were excluded.

This study was approved by the Human Research Ethics Committee at the institution where the research was carried out (registration no. CAAE 74449317.1.0000.5417; September 11, 2017).

### Data collected and study group characteristics

The following data on demographic and treatment characteristics were collected from the hospital’s electronic medical records: age, sex, diagnosis of cancer, evolution of cancer, comorbidities, complete blood count and chemotherapy regimen. These regimens included FOLFOX (oxaliplatin + calcium folinate + fluorouracil), TAXOL (paclitaxel), MTX (methotrexate), Gemzar, FOLFIRI (folinic acid, fluorouracil and irinotecan), CARBO-TAXOL (carboplatin + paclitaxel), 5-FU (fluorouracil) and others (vinorelbine, ifosfamide, etoposide, cisplatin, capecitabin, bicalutamide, vincristine, vinblastine, irinotecan, cyclophosphamide and doxorubicin).

The following data from the dental evaluation were collected: oral regions with OM, classification of the OM and oral health of the individuals during hospitalization.

Individuals with OM were evaluated with regard to the number of oral regions affected, presence of erythema and ulcers, duration of manifested OM and oral pain (assessed using a VAS).^10^ OM was classified in accordance with the World Health Organization categories (OM-WHO) and the modified WHO scale (mOM-WHO).^11,12^ To assess the oral health involvement of the individuals included in this study, we used the Bedside Oral Examination (BOE), which classifies the oral condition as normal oral condition (score of 8 to 10), moderately impaired oral condition (score of 11 to 14) or very impaired oral condition (score of 15 to 24).^13^

All individuals seen by an oncologist were also evaluated with regard to their need for dental evaluation. Through this perception, consultations were requested by the dental team during these patients’ hospitalization. All individuals examined by dentists were under dental care during hospitalization and received the same treatment for OM.

The therapeutic strategies used in relation to OM depended on the degree of OM and its associated comorbidities, such as hypertension, diabetes and other conditions. For grades 1 and 2, low-power laser therapy at 660 nm (100 mW) with E = 2 J, at a dose of 20 J/cm^2^, was used. For grades 3 and 4, the same laser therapy dose with benzydamine hydrochloride (1.5 mg/ml) was used. In cases in which a focus of infection was found associated with OM grade 1 and 2, 0.12% chlorhexidine without alcohol administered every 12 hours was prescribed; and in cases of OM grades 3 and 4, this topical antibiotic was indicated for administration between meals. In the presence of labial dryness in cases of OM grades 1 and 2, *Chamomilla recutita* Rauschert extract (100 mg) was applied; in cases of OM grades 3 and 4, it was applied after laser therapy and before intraoral manipulation.

Transient myelosuppression was evaluated by analyzing the complete blood count performed at the time of the most severe OM. Red blood cell, leukocyte, neutrophil and platelet counts were also evaluated. The degree of myelosuppression and the respective ratings and references for men and women are shown in Table 1.

**Table 1:** Reference values for transient myelosuppression

Anemia	Hemoglobin Men	Hemoglobin Women	Thrombocytopenia	Platelets
Grade I	≥ 13	≥ 12	Grade I	≥ 151,000
Grade II	10.1-12.9	10.1-11.9	Grade II	81000-150,000
Grade III	9.1-10	9.1-10	Grade III	51,000-80,000
Grade IV	7.1-9	7.1-9	Grade IV	31,000-50,000
Grade V	0-7	0-7	Grade V	0-30,000
**Leukopenia**	Leukocytes		Neutropenia	Neutrophils
Grade I	≥ 3,501		Grade I	≥ 2,001
Grade II	2,001-3,500		Grade II	1,001-2,000
Grade III	1,001-2,000		Grade III	501-1,000
Grade IV	0-1,000		Grade IV	0-500

### Statistical analysis

To analyze the data distribution, the Shapiro-Wilk test was applied. Descriptive analysis was performed based on the prevalence and average. Comparisons between variables were analyzed using the Mann-Whitney and Kruskal-Wallis tests, and correlations were performed using Spearman’s correlation. The level of significance was set at 5% (P < 0.005).

## RESULTS

### Patients’ demographic data and characteristics

A total of 115 medical records were evaluated. These individuals had a mean age of 47.5 years (range: 2 to 90 years) and comprised 63 males (54.8%) and 52 females (45.2%). Gastric tumors (33.9%) and osteosarcoma (15.7%) were the most prevalent conditions, and the most prevalent chemotherapy protocols were MTX (12.2%) and FOLFOX (12.2%). The prevalences of other tumors and certain chemotherapy protocols are shown in Tables 2 and 3, respectively.

**Table 2:** Prevalence of solid tumors (n = 115)

Diagnosis	n (%)
Gastric tumors	39 (33.9%)
Osteosarcoma	18 (15.7%)
Breast cancer	15 (13.01%)
Lung cancer	12 (10.4%)
Urinary tract cancer	11 (9.6%)
Gynecological cancer	6 (5.2%)
Tumors of the nervous system	4 (3.5%)
Others	10 (8.7%)

**Table 3:** Prevalence of chemotherapy protocols (n = 115)

CT protocol	n (%)
MTX	16 (13.9%)
FOLFOX	14 (12.2%)
Gemcitabine hydrochloride	13 (11.3%)
FOLFIRI	10 (8.7%)
Taxol + Carbo	7 (6.1%)
5-FU	6 (5.2%)
Others	49 (42.6%)

MTX = methotrexate; FOLFOX = oxaliplatin + calcium folinate + fluorouracil; gemcitabine hydrochloride = gemcitabine + diphenhydramine; FOLFIRI = irinotecan + fluorouracil + calcium folinate; Taxol + Carbo = carboplatin + paclitaxel; 5-FU = fluorouracil.

No therapeutic measures that could potentiate OM were used. Out of the 115 patients, 24 underwent associated radiotherapy treatment. Of these, only one patient underwent radiotherapy in the head and neck region, while the others had indications for the lower respiratory tract, prostate, uterus, breast, pelvis and digestive tract regions.

### Oral mucositis

Regarding OM, 28 individuals (24.3%) presented with some clinical manifestations of OM. The most prevalent degrees of OM were degree 2 (11.3%) according to the OM-WHO classification and grades 1 and 5 according to mOM-WHO (4.3%) (Table 4).

**Table 4:** Prevalence severity of OM-WHO and mOM-WHO

Degree	WHO mucositis	Modified WHO mucositis
0	87 (75.6%)	87 (75.6%)
1	7 (6.1%)	5 (4.3%)
2	13 (11.3%)	4 (3.5%)
3	7 (6.1%)	4 (3.5%)
4	1 (0.9%)	5 (4.3%)
5	NA	10 (8.7%)

OM-WHO = oral mucositis according to the World Health Organization classification; mOM-WHO = oral mucositis according to the modified World Health Organization classification; NA = not applicable.

**Figure 1:**
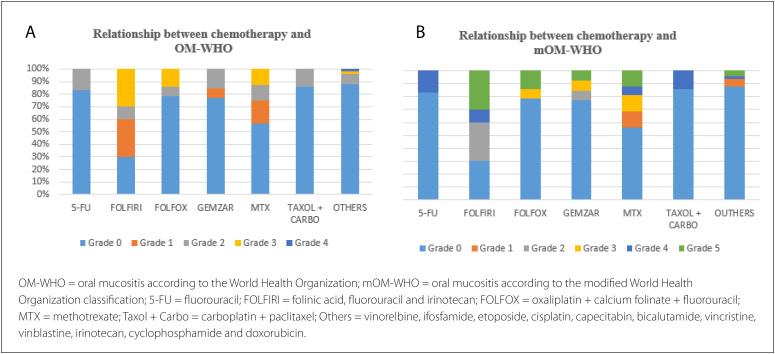
Relationship between chemotherapy protocols and OM prevalence on the OM-WHO and mOM-WHO scales.

It was observed that the regions most affected by OM (Table 5) were the tongue, lower lip, upper lip and buccal mucosa in equal proportions (64.3%). Moreover, OM was manifested in these regions primarily when the chemotherapy protocols used were FOLFIRI, FOLFOX, MTX and gemcitabine hydrochloride. Higher degrees of OM-WHO (P = 0.009) and mOM-WHO (P = 0.004) were observed when the FOLFIRI protocol was used for AnT. Figure 1 shows the relationship between chemotherapy protocols and the prevalence of OM.

**Table 5:** Prevalence of oral regions with oral mucositis

Oral regions	n (%)
Language	18 (64.3%)
Bottom lip	18 (64.3%)
Jugal mucosa	18 (64.3%)
Upper lip	14 (50%)
Soft palate	5 (17.8%)
Hard palate	3 (10.7%)
Gum	2 (7.1%)
Alveolar mucosa	2 (7.1%)
Oral floor	1 (3.6%)
Throat	1 (3.6%)
Retromolar region	0 (0%)

With regard to the ratio between OM and pain, the higher the degrees of OM-WHO (P < 0.001; r = 0.306) and mOM-WHO were (P < 0.001; r = 0.295), the higher the level of mouth pain reported by individuals was. In addition, manifestation of OM on the upper lip (P < 0.044) and jugal mucosa (P = 0.005) contributed to increased severity of OM-WHO.

### Mouth condition

In the BOE assessment, 58 individuals (50.4%) had a normal oral condition, 53 (46.3%) had a moderately impaired oral condition and only 4 (3.5%) had a very impaired oral condition.

By analyzing the relationship between oral conditions and OM, it was observed that worse oral condition was significantly associated with greater degrees of OM-WHO (P = 0.025; r = 0.208) and mOM-WHO (P < 0.001; r = 0.228).

**Figure 2:**
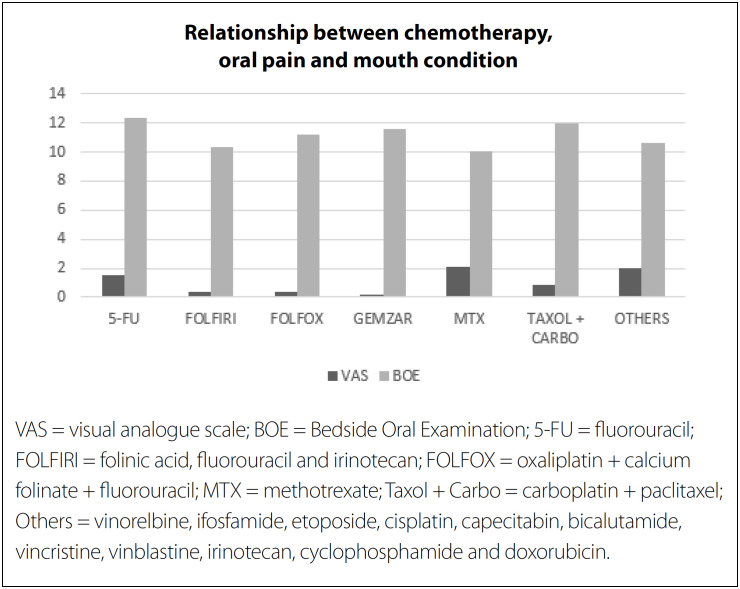
Relationship between chemotherapy protocols and oral pain (from visual analogue scale, VAS) and oral mouth condition (from bedside oral examination, BOE).

The oral condition of individuals who received certain types of chemotherapy was significantly correlated with more severe manifestations of OM-WHO (P = 0.025; r = 0.208) and mOM-WHO (P < 0.001; r = 0.228). Individuals under the 5-FU regimen presented worsening of their oral condition (BOE) (P = 0.038), especially when OM was present in the lips, tongue and gums. Figure 2 shows the relationship between chemotherapy protocols and VAS and BOE.

### Evaluation of myelosuppression

**Figure 3:**
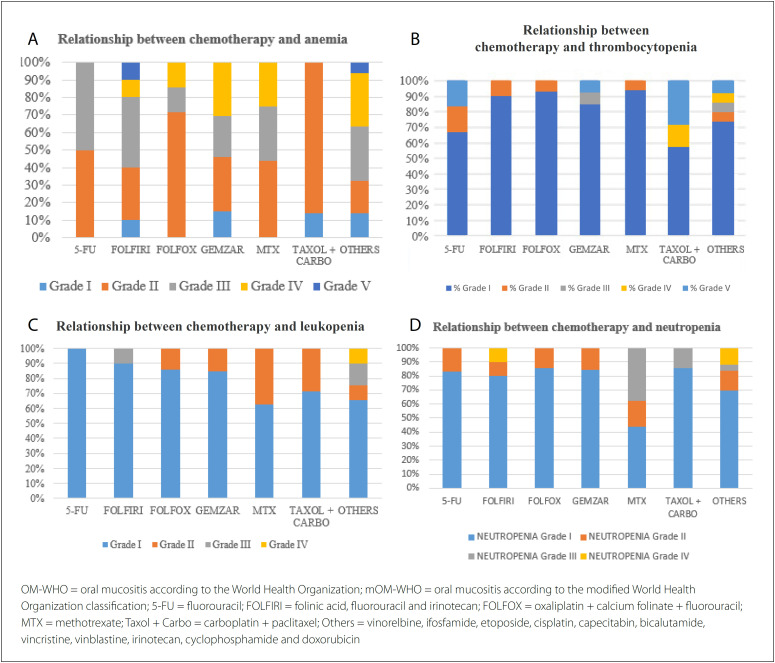
Relationship between chemotherapy protocols and transient myelosuppression.

Complete blood count examinations revealed that 104 patients (90.44%) had anemia, 23 (19.93%) had thrombocytopenia, 30 (26.09%) had leukocytosis and 32 (27.83%) had neutropenia (Table 6). However, there were no significant correlations between specific chemotherapy protocols and the severity of OM-WHO and mOM-WHO. Nor were there any statistically significant correlations between chemotherapy protocols and myelosuppression reference values (P > 0.05). Figure 3 shows four graphs correlating the chemotherapy protocols with anemia, thrombocytopenia, leukopenia and neutropenia.

**Table 6:** Prevalence of myelosuppression (n = 115)

Anemia	n (%)	Thrombocytopenia	n (%)
Grade 0	11 (9.56%)	Grade 0	92 (80.0%)
Grade 1	42 (36.52%)	Grade 1	7 (6.09%)
Grade 2	32 (27.83%)	Grade 2	4 (3.48%)
Grade 3	26 (22.61%)	Grade 3	4 (3.48%)
Grade 4	4 (3.48%)	Grade 4	8 (6.96%)
**Leukopenia**		**Neutropenia**	
Grade 0	85 (73.91%)	Grade 0	83 (72.17%)
Grade 1	17 (14.78%)	Grade 1	16 (13.91%)
Grade 2	8 (6.96%)	Grade 2	9 (7.83%)
Grade 3	5 (4.35%)	Grade 3	7 (6.09%)

## DISCUSSION

Individuals under AnT for solid tumors have risk factors that contribute to manifestation of severe OM. These factors may be related to systemic conditions, such as certain types of chemotherapy (CT); or to local conditions, such as damage to the oral cavity. In the present study, a relationship was observed between damage to the oral cavity and manifestation of severe OM.^14,15^

Due to the intense discomfort, buccal pain and significant worsening of oral condition in relation to OM among individuals under hospitalization for AnT, it is important and necessary to trace individuals who are at higher risk of OM.^16^

In this study, OM was observed in 24.3% of the individuals. This proportion was similar to what was found in other studies that evaluated OM in individuals who underwent certain types of chemotherapy for solid tumors. In one such study, the prevalence of OM was 24% among individuals who underwent a second cycle of certain types of chemotherapy; and in another, it was 31% under the same treatment.^17,18^

When 5-FU is used in protocols such as TPF, CAF or FOLFIRI, the incidence rate of OM can reach more than 15%, causing OM-WHO grade 3-4. That incidence rate was similar to the result from our study (16.7%), but the severity differed according to the scale: OM-WHO scale, grade 2, versus mOM-WHO scale, grade 4.^19^

The use of 5-FU worsens oral health, especially in the lips, tongue and gums, considering that these are the mouth regions most affected. When 5-FU was used, the risk of developing severe OM-WHO was found to be 15% higher, consequently worsening the patients’ swallowing capacity.^20^ The oral health of patients under 5-FU treatment can become worse because, aside from the oral pain caused by OM, these individuals also experience constant nausea and vomiting.^21^ In addition, hyposalivation in individuals under 5-FU treatment increases the incidence of mucositis, which suggests that this is a risk factor for OM.^22–25^

Another important symptom, which was also observed in this study, was oral pain. This is a fundamental issue that interferes with the quality of life of individuals with cancer, and it is directly related to severe OM.^16^ It has been observed in other studies that patients on AnT for solid tumors who develop OM have worse quality of life than those without OM, and that this manifestation predisposes them to other side effects such as pain and poor physical and emotional wellbeing, which negatively impacts their quality of life.^17^

Understanding the risk factors relating to OM and the oral conditions that can compromise quality of life and interrupt AnT is necessary. Oral condition is directly related to the severity and repair of OM.^15,26^ In previous studies, worse oral condition was related to greater severity of OM. However, those studies did not find any significant relationship between dental and prosthetic conditions and the severity of OM, as found in our study. One possible explanation for this is that although the visible plaque index and gingival plaque index are directly related to the incidence of OM, the ratio of lost, decayed or restored teeth was not determined by those authors.^26^

There is evidence that neutropenic patients are between three and as much as 7.5 times more likely to develop OM than are patients without neutropenia.^27,28^ In our study, we analyzed the correlation between the severity of OM and use of certain chemotherapy protocols and transient myelosuppression, but no significant result was found. In a study on oncopediatric patients under hospitalization, a significant relationship was observed, and this was explained by the degree of neutropenia, which was shown to influence the risk of developing OM, and by the difference in the QT protocols applied.^28^

This study brought a lot of relevant information relating to the severity of oral mucositis, with regard to oral pain and chemotherapy protocols for oral health. However, it should be considered that this was a cross-sectional study, consisting of analysis on the medical records of individuals hospitalized under a single cycle, which limited assessment of the cumulative effect of chemotherapy cycles on the severity of oral mucositis. In addition, no data on tumor staging, drug doses used or other drugs used concomitantly that could directly interfere with oral toxicity were collected. Lastly, other limitations of this study that may have confounded the analysis on the results comprised the small sample, wide age range and heterogeneity of the study group.

## CONCLUSIONS

Individuals with solid tumors who presented with severe OM had greater severity of oral pain and worse oral health. Use of the FOLFIRI chemotherapy protocol was associated with higher prevalence of severe OM. Individuals who used 5-FU had worse oral condition, mainly with regard to changes to the lips, tongue and gums. Knowledge of chemotherapy protocols helps identify individuals with a greater chance of developing severe OM or with a worse oral condition through use of certain types of chemotherapy during hospitalization. Through screening, it is possible to reduce morbidity and mortality and provide better quality of life for patients with cancer.
